# Anticoagulants for stroke prevention in heart failure with reduced ejection fraction

**DOI:** 10.1007/s00392-021-01930-y

**Published:** 2021-08-27

**Authors:** Andreas Schäfer, Ulrike Flierl, Johann Bauersachs

**Affiliations:** Department of Cardiology and Angiology, Hannover Medical School, Carl-Neuberg-Str. 1, 30625 Hannover, Germany

**Keywords:** Anticoagulation, VKA, NOAC, Heart failure, Rivaroxaban

## Abstract

Impaired left-ventricular ejection-fraction (LV-EF) is a known risk factor for ischemic stroke and systemic embolism in patients with heart failure (HF) even in the absence of atrial fibrillation. While stroke risk is inversely correlated with LV-EF in HF patients with sinus rhythm, strategies using anticoagulation with Vitamin-K antagonists (VKA) were futile as the increase in major bleedings outweighed the potential benefit in stroke reduction. Non-Vitamin K oral anticoagulants (NOACs) proved to be an effective and in general safer approach for stroke prevention in patients with atrial fibrillation and may also have a favourable risk–benefit profile in HF patients. In HF patients with sinus rhythm, the COMPASS trial suggested a potential benefit for rivaroxaban, whereas the more dedicated COMMANDER-HF trial remained neutral on overall ischemic benefit owed to a higher mortality which was not influenced by anticoagulation. More recent data from subgroups in the COMMANDER-HF trial, however, suggest that there might be a benefit of rivaroxaban regarding stroke prevention under certain circumstances. In this article, we review the existing evidence for NOACs in HF patients with atrial fibrillation, elaborate the rationale for stroke prevention in HF patients with sinus rhythm, summarise the available data from anticoagulation trials in HF with sinus rhythm, and describe the patient who might eventually profit from an individualised strategy aiming to reduce stroke risk.

**Graphic abstract:**

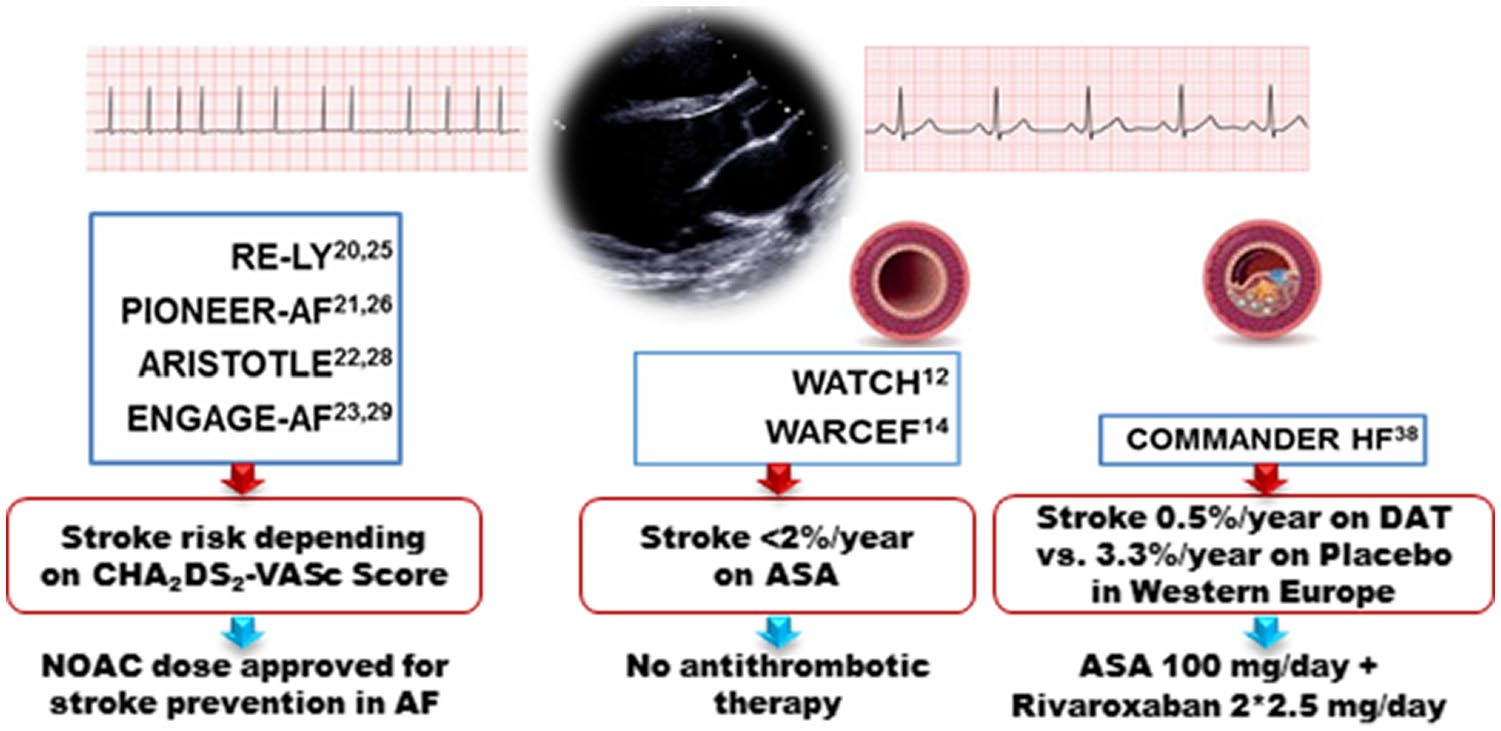

## A typical clinical scenario illustrating the dilemma

A 40-year-old male Caucasian was resuscitated due to ventricular fibrillation. The patient was admitted, a 12-lead ECG showed sinus rhythm and absence of ST-elevation. Coronary angiography excluded pre-existing coronary artery disease. Transthoracic echocardiography revealed global hypokinesia with a left-ventricular ejection fraction (LV-EF) of 20%. The patient was treated with an implantable cardioverter-defibrillator and all subsequent controls exclude irregular atrial activity. Fearing an increased stroke risk due to severely reduced LV function in heart failure (HF) with sinus rhythm, the patient’s cardiologist initiated oral anticoagulation (OAC) using phenprocoumon (at a time when this was the only oral anticoagulation available) with a target international normalised ratio (INR) of 2–3. After 3 years of event-free survival and continued impaired LV-EF of 20%, the patient’s cardiologist retired and his new cardiologist stopped OAC and initiated platelet inhibition using acetylsalicylic acid (ASA, 100 mg/day). 5 months later, the patient was urgently admitted with hemiplegia due to thromboembolic stroke owing to a large LV thrombus (LV-EF 20%). Was it correct to stop OAC in HF with reduced ejection fraction (HFrEF)? Was OAC indicated in the first place? What kind of OAC might provide clinical benefit in modern times?

## Increased stroke risk in patients with chronic heart failure and sinus rhythm

During the 1990s, large heart failure trials such as Survival and Ventricular Enlargement (SAVE) and Studies of Left Ventricular Dysfunction (SOLVD) indicated an increased risk of thromboembolism with decreasing LV-EF postulating an inverse relation of 18% relative increase in stroke per 5% absolute reduction in LV-EF [1–3]. In a nationwide Danish cohort study including almost 43.000 patients, of whom only 22% had atrial fibrillation, HF patients had a high ischemic stroke risk, the CHA_2_DS_2_-VASc score modestly predicted the risk, and patients with HF in the absence of atrial fibrillation and CHA_2_DS_2_-VASc scores ≥ 4 had a high absolute risk of ischemic stroke. HF patients without atrial fibrillation and high risk scores had a similar rate of thromboembolism as patients with atrial fibrillation [4]. In a recent report from the Swedish Heart Failure Registry, 15.425 patients with HF and sinus rhythm were compared to 28.815 age- and sex-matched controls without HF from the Swedish Population Register. The 2-year stroke rate in HF with sinus rhythm compared to controls was 1.4% vs. 0.4% for patients < 65 years of age, 3.0% vs. 1.2% for patients aged 65–74 years, 4.0% vs. 2.2% for patients aged 75–84 years, and 3.9% vs. 2.3% for patients aged > 84 years. All-cause mortality after two years in the HF groups was reported as 8.1%, 17.0%, 30.4%, and 53.0%, respectively [5]. While the risk of stroke has been determined in multiple analyses, current treatment of comorbidities in HF focus on non-embolic diseases [6]. Regarding the case scenario for an HF patient in SR, existing evidence suggests a relevant stroke rate in this condition.

## Pathophysiology of stroke in heart failure

There are several potential contributors to stroke risk in HF, which can be divided into embolic and haemodynamic. For embolic stroke, three potential scenarios are likely to contribute. First, clinically unapparent atrial fibrillation might cause thrombus formation in the left atrial appendage. Second, severely impaired LV function might lead to formation of LV thrombus. Third, endothelial dysfunction, which is common in HF, can contribute to atherothrombosis in supra-aortic arteries. Recent retrospective analyses suggest that anticoagulant non-vitamin K oral anticoagulant (NOAC) strategies comparably effective to VKA in reducing stroke rates in AF patients might not be as efficient for stroke prevention due to LV thrombus [7]. While these potential sources for embolic strokes appear intuitively as useful targets for anticoagulants, cerebral hypoperfusion due to impaired haemodynamics in heart failure causing symptoms comparable to stroke may not be reduced by anticoagulation. In HF, all components of the Virchow’s triad comprising endothelial dysfunction, hypercoagulability, and impaired blood flow are negatively affected. However, anticoagulation will more or less only address the one caused by hypercoagulability. We will now focus on the potential improvement of this imbalance by using OAC with lower bleeding rates than reported for VKA. Nevertheless, the pathophysiological considerations pointed out above already indicate why it may be difficult to achieve a significant reduction of stroke or stroke-like symptoms in HF patients by anticoagulation.

## Guideline recommendations for HFrEF patients in sinus rhythm

Current European and American guidelines recommend that patients with HFrEF receiving OAC because of concurrent AF or risk of venous thromboembolism should continue anticoagulation [8, 9]. Other than in patients with AF (both for reduced as well as for preserved EF), evidence that an oral anticoagulant reduces mortality or morbidity compared with placebo or ASA is lacking [8, 9]. Similarly, there is no recommendation on antiplatelet drugs (including ASA) in patients with HF without accompanying coronary artery disease, based on lacking evidence of stroke reduction but substantial risk of gastrointestinal bleeding, particularly in elderly subjects [8]. Regarding the case scenario for an HF patient in SR, guidelines do neither recommend anticoagulation nor platelet inhibition in this condition due to lacking evidence.

## Evidence for VKA to reduce stroke risk in HF with sinus rhythm

Descriptive information for trials assessing the potential impact of VKA on stroke prevention in HF patients with sinus rhythm are provided in Table 1. The Heart failure Long-term Antithrombotic Study (HELAS) compared double-blinded warfarin to placebo (ASA 325 mg/day in case of previous myocardial infarction). Due to the small number of patients enrolled, it was not possible to evaluate differences in efficacy between the treatment groups, e.g. regarding the endpoint of stroke, which occurred in five patients (two on warfarin, three on ASA/placebo) [10].

**Table 1:** Characteristics of Vitamin K oral anticoagulant trials for potential stroke prevention in heart failure

	HELAS [10]	WASH [11]	WATCH [12]	PRIME II [13]	WARCEF [14]
Comparator to VKA	ASA/placebo	No antithrombotic	ASA/clopidogrel	OAC or antiplatelet to no antithrombotic	ASA
INR target	2.0–3.0	2.0–3.0	2.0–3.0	Retrospective, no target	2.0–3.5
Patients (*n*)	312	279	1587	427	2305
Definition of HF	Previous MI or DCM	HF with LVSD	Reduced LV-EF	Advanced HF (LVEDD > 60 mm, LV-EF < 35%, or cardiothoracic ratio > 0.5 on chest X-ray)	Reduced LV-EF
Definition of reduced LV-EF	≤ 35%	≤ 35%	≤ 35%	< 35%	≤ 35%
Mean follow-up (years)	1.6	2.3	1.9	5	3.5
Endpoint analysis	No	No	No	No	Yes

*ASA* acetyl-salicylic acid, *DCM* dilated cardiomyopathy, *HF* heart failure, *INR* international normalised ratio, *LVEDD* left-ventricular end-diastolic diameter, *LV-EF* left-ventricular ejection fraction, *LVSD* left-ventricular systolic dysfunction (defined as increased left ventricular end-diastolic internal dimension (56 mm or 30 mm/m^2^ body surface area), *MI* myocardial infarction, *VKA* vitamin K antagonist

The Warfarin/Aspirin Study in Heart failure (WASH) compared no antithrombotic therapy with open-label warfarin. Again, based on the small sample size, no sound recommendation could be made regarding the endpoint of stroke, which occurred in two patients (none on warfarin, one on ASA, and one without antithrombotic treatment) [11].

The Warfarin and Antiplatelet Therapy in Chronic Heart Failure trial (WATCH) compared open-label warfarin with to double-blinded treatment with either ASA (162 mg/day) or clopidogrel (75 mg/day. The trial had been terminated prematurely because of slow enrolment (intended sample size 4.500). In WATCH, stroke rate was reduced from 2.3% on ASA or clopidogrel (without any difference between both) to 0.6% on VKA, but major bleedings increased from 3.6% on ASA and 2.1% on clopidogrel to 5.2% on VKA (*p* = 0.0074 for clopidogrel vs. VKA, *p* = 0.2184 for ASA vs. VKA). However, the net clinical benefit consisting of bleeds in the central nervous system or stroke was non-significantly lower on VKA (1.7%) compared to ASA (2.9%) and clopidogrel (2.5%) [12].

A retrospective analysis of the Prospective Randomised study of Ibopamine on Mortality and Efficacy (PRIME-II) conducted between 1992 and 1995 in the Netherlands assessed the potential influence of concomitant antithrombotic therapy. In this retrospective analysis, stroke rate or bleedings have not been reported, but the analysis suggested lower mortality in HF patients with sinus rhythm using OAC compared to those not on OAC [13].

The Warfarin versus Aspirin in Reduced Cardiac Ejection Fraction (WARCEF) Study Group compared double-blinded warfarin to ASA. In this largest trial assessing the potential superiority of VKA over ASA, the stroke rate was reduced from 1.36 per 100 patient-years on ASA to 0.72 per 100 patient-years on VKA (*p* = 0.005; absolute difference 26 events). The rate of intracerebral haemorrhage increased from 0.05 per 100 patient-years on ASA to 0.12 per 100 patient-years on VKA (*p* = 0.005; absolute difference 3 events). The rate of major bleedings in general increased from 2.7% on ASA to 5.8% on VKA (*p* < 0.001; absolute difference 35 events) mainly driven by an increase in gastrointestinal bleeding. In some in-depth analysis, there appeared to be a potential benefit of VKA of “uncertain clinical significance” among patients followed up for 4 years or more[14].

In summary, stroke rates higher than 3% per year have been considered to justify anticoagulation with VKA for ischemic benefit to outweigh bleeding risk in patients with atrial fibrillation in the past [15]. The existing evidence neither suggests a relevantly increased annual stroke risk in HF patients in sinus rhythm nor does it show a statistically significant benefit for VKA compared to ASA regarding stroke prevention in HF patients with impaired systolic function when these patients remained in sinus rhythm [16, 17]. Regarding the case scenario for an HF patient in SR, existing evidence suggests no benefit for anticoagulation with VKA in this condition.

## Evidence for NOAC to reduce stroke risk in HF with atrial fibrillation

**Fig. 1:**
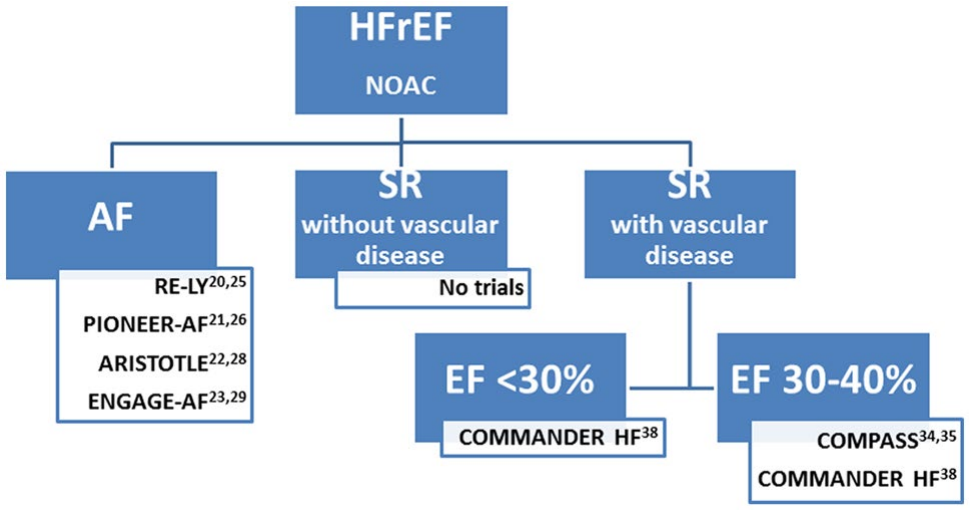
Clinical trials reporting stroke risk in patients with heart failure depending on the extent of left-ventricular impairment and coexistent vascular disease or atrial fibrillation; AF, atrial fibrillation; (LV-)EF, left-ventricular ejection fraction; NOAC, Non-vitamin K oral anticoagulants; SR, sinus rhythm

**Fig. 2:**
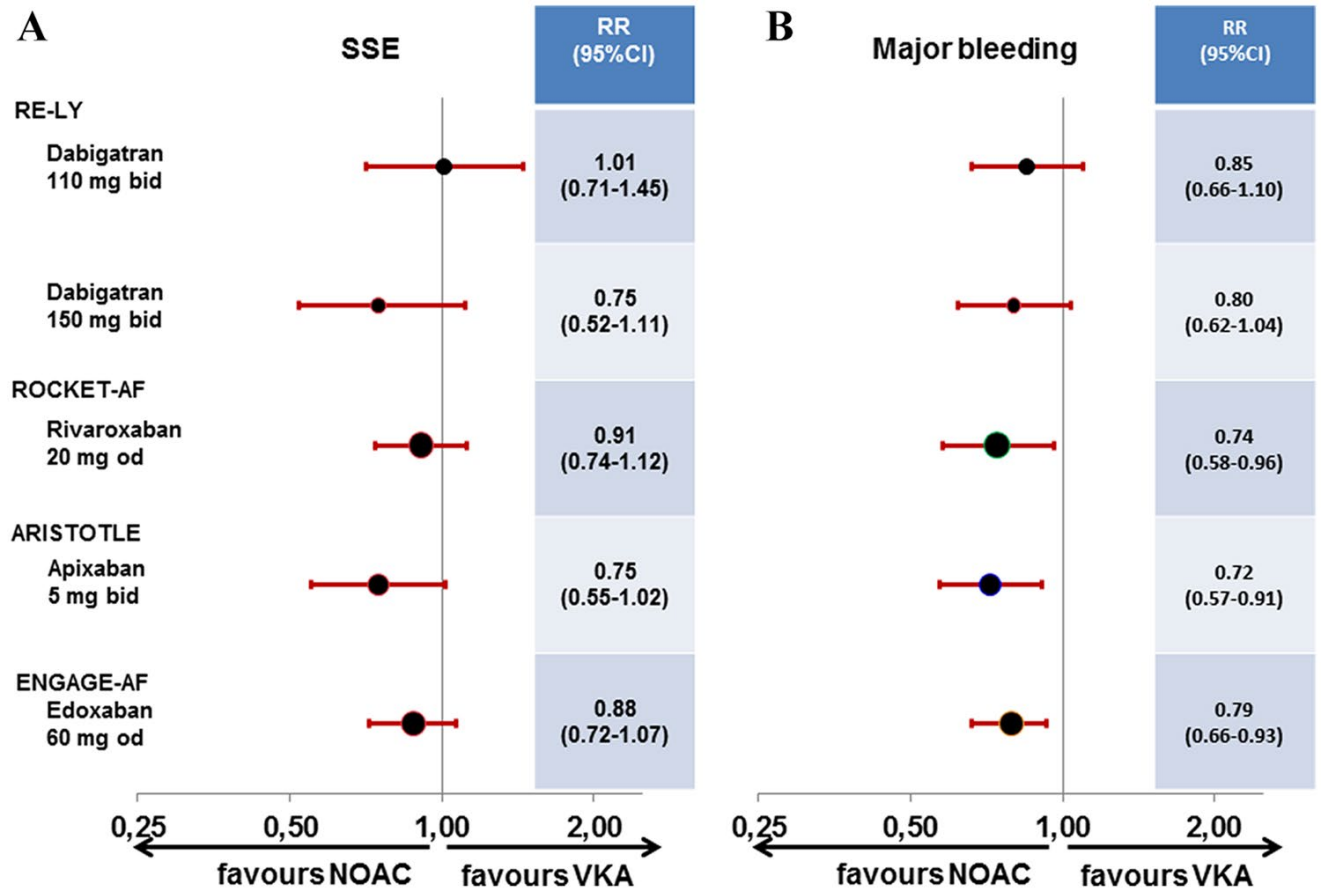
Relative risk for stroke or systemic embolism **a** and major bleeding **b** in patients with heart failure in the four trials comparing Non-vitamin K oral anticoagulants to Vitamin K-antagonist for stroke prevention in atrial fibrillation; *OR* odds ratio, *NOAC* non-vitamin K oral anticoagulants, *SSE* stroke or systemic embolism, *VKA* vitamin K-antagonist. Definition of major bleeding was according to study criteria [21–24]

**Fig. 3:**
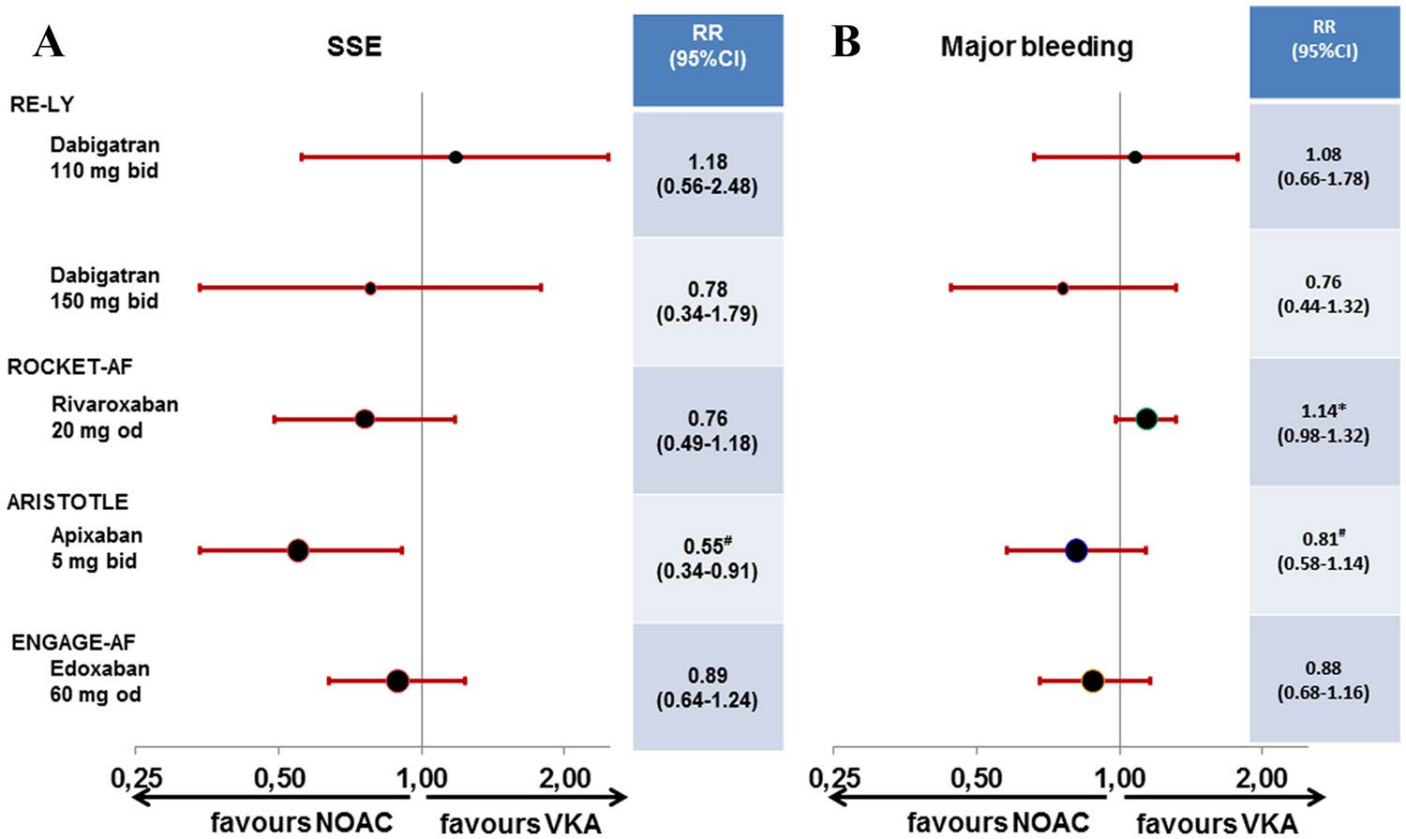
Relative risk for stroke or systemic embolism **a** and major bleeding **b** in patients with heart failure and reduced LV ejection fraction in the four trials comparing non-vitamin K oral anticoagulants to Vitamin K-antagonist for stroke prevention in atrial fibrillation; *OR* odds ratio, *NOAC* non-vitamin K oral anticoagulants, *SSE* stroke or systemic embolism, *VKA* vitamin K-antagonist. Definition of major bleeding was according to study criteria [21–24].*RR calculated based on the data for major and clinically-relevant non-major bleeding [28]; ^#^individual data not published, data represents the hazard ratios reported for the respective subpopulation [30]

Using newer and safer oral anticoagulants, lower stroke rates as low as approximately 1% per year are now considered as an potential indication for OAC in patients with atrial fibrillation if the bleeding risk is sufficiently low [18]. However, NOACs have only been investigated in HFrEF patients with either coexistent vascular disease or atrial fibrillation (Fig. 1). In patients with atrial fibrillation, there is at least some evidence indicating a comparable bleeding risk for apixaban and ASA [19, 20]. In the AF approval trials [21–24], NOACs in general were safer and more effective than VKA also in high-risk patients such as the elderly [25]. In the overall HF subgroups, all four NOACs were at least equally effective or better in preventing stroke or systemic embolism, and factor Xa-inhibitors in particular demonstrated lower rates of major bleeding compared to VKA (Fig. 2) [26]. The differences in definition of HF as well as detailed information on event rates in HF subgroups and those with reduced LV-EF are provided in Table 2; event rates for dedicated subgroups are listed in Table 3. The calculated relative risk for embolism and major bleedings are displayed for the overall HF subgroups (Fig. 2) and for the specific subgroups with reduced LV-EF (Fig. 3).

**Table 2:** Characteristics of HF populations in non-vitamin K oral anticoagulant approval trials for stroke prevention in atrial fibrillation

	RE-LY [21]	ROCKET-AF [22]	ARISTOTLE [23]	ENGAGE-AF [24]
Comparator to VKA	Dabigatran	Rivaroxaban	Apixaban	Edoxaban
Standard dose	150 mg twice daily 110 mg twice daily	20 mg once daily	5 mg twice daily	60 mg once daily
Reduced dose	–	15 mg once daily	2.5 mg twice daily	30 mg once daily
Dose reduction criteria	1:1:1 randomised to either dose or VKA	creatinine clearance 30–49 mL/min	at least two criteria:	At least one criterion:
			Age ≥ 80 years	Estimated creatinine clearance 30–50 mL/min
			Body weight < 60 kg	Body weight ≤ 60 kg
			Serum creatinine ≥ 1.5 mg/dL	concomitant use: ciclo-sporine, dronedarone, ery-thromycin, or ketoconazole
Patients with heart failure (*n*)	4904 (27% of trial patients)	9033 (64% of trial patients)	5943 (33% of trial patients)	8145 (58% of trial patients)
Definition of heart failure	Presence of NYHA class ≥ II symptoms (fatigue, dyspnoea) in the 6 months prior to screening in patients with a history of previous admission for congestive HF. Information on LV-EF was only available in 59% of HF patients	History of HF or LV-EF < 40%	symptomatic congestive HF within 3 months or LV-dysfunction with an EF ≤ 40%	previous history of HF stage C or D according to AHA/ACC. LV-EF was available in 79% of HF patients
Patients with reduced LV-EF	1258 (26% of HF population)	2497 (27% of HF population)	2736 (46% of HF population)	3103/21,105 (38% of HF population)
Definition of reduced LV-EF	≤ 40%	< 40%	≤ 40%	< 50%
Mean age in HF population (years)	68	72	69	70
CHADS_2_ in HF population	2.6 ± 1.1	3.7 ± 0.9	2.4 ± 1.1	3.0 ± 1.0
Definition of primary safety endpoint	Major bleeding by study definition [21]	Composite of major and non-major clinically relevant bleeding by study definition [22]	Major bleeding defined by ISTH criteria [29]	Major bleeding defined by ISTH criteria [29]

*CHADS*_*2*_ score to predict annual stroke risk in atrial fibrillation [45], *HF* heart failure, *ISTH* International Society on Thrombosis and Haemostasis, *LV-EF* left-ventricular ejection fraction, *VKA* vitamin K antagonist

**Table 3:** Outcome of HF populations in Non-Vitamin K oral anticoagulant approval trials for stroke prevention in atrial fibrillation

	RE-LY [21] dabigatran	ROCKET-AF [22] rivaroxaban	ARISTOTLE [23] apixaban	ENGAGE-AF [24] edoxaban
Rate for stroke/systemic embolism in HF vs non-HF, HR (95% CI)	1.75 vs 1.35%/year 1.08 (0.89–1.31)	1.99 vs 2.32/100 p-y 0.94 (0.78–1.13)	HF-PEF: 1.52 vs 1.37/100 p-y 1.11 (0.87–1.42)	NYHA I-II:1.62 vs 1.66%/year 1.19 (0.99–1.42)*
			LVSD: 1.39 vs 1.37/100 p-y 1.01 (0.77–1.33)	NYHA III-IV:2.00 vs 1.66%/year 1.45 (1.12–1.88)*
Rate for primary safety event in HF vs non-HF, HR (95% CI)	3.42 vs 3.19%/year 1.03 (0.90–1.17)	14.12 vs 15.73/100 p-y 1.00 (0.92–1.08)	HF-PEF: 2.55 vs 2.50/100 p-y 1.02 (0.84–1.24)	NYHA I-II:2.96 vs 3.31%/year 1.24 (1.07–1.43)*
			LVSD: 3.09 vs 2.50/100 p-y 1.23 (1.01–1.50)	NYHA III-IV:2.83 vs 3.31%/year 1.31 (1.05–1.65)*
Rate for stroke/systemic embolism vs VKA in patients with reduced LV-EF, HR (95% CI)	110 mg: 1.89 vs 1.61%/year 1.18 (0.55–2.53)	1.34 vs 1.87/100 p-y 0.72 (0.46–1.12)	0.99 vs 1.80/100 p-y^#^ 0.55 (0.34–0.91)	1.55 vs 1.76%/year 0.89 (0.63–1.24)
	150 mg: 1.23 vs 1.61%/year 0.76 (0.33–1.76)			
Rate for primary safety vs VKA in patients with reduced LV-EF, HR (95% CI)	110 mg: 3.91 vs 3.62%/year 1.09 (0.65–1.83) 150 mg:	15.34 vs 14.10/100 p-y 1.15 (0.96–1.36)	2.77 vs 3.41/100 p-y^#^ 0.81 (0.58–1.14)	2.87 vs 3.21%/year 0.91 (0.69–1.20)
	2.70 vs 3.62%/year 0.75 (0.43–1.32)			

HF, heart failure; HR, hazard ratio (*HRs in ENGAGE-AF had been adjusted for unbalanced co-variates by the original investigators [31];^#^event rates and HRs for LV-EF subpopulation in ARISTOTLE are shown as published by the original investigators [30]); LV-EF, left-ventricular ejection fraction; p-y, patient-years; VKA, Vitamin K antagonist

### Dabigatran in RE-LY

The RE-LY trial compared the direct thrombin-inhibitor dabigatran (110 mg and 150 mg twice daily) to warfarin [21]. The trial included 4904 patients with HF. Information on LV-EF were only available in 59% of HF patients. Irrespective of randomisation, embolic events as well as the rate of major bleedings were numerically higher in patients with HF. In the 1258 HF patients with known reduced LV-EF, compared to warfarin the dabigatran 110 mg dose was associated with numerically higher rates of embolism and major bleeding, while he dabigatran 150 mg dose was associated with numerically lower rates of embolism and major bleeding [27]. While the numbers of patients with confirmed HFrEF are too low and too much data are missing to provide solid conclusions, the available data in HFrEF patients with atrial fibrillation suggests potential non-inferiority in patients on dabigatran 150 mg twice daily.

### Rivaroxaban in ROCKET-AF

The ROCKET-AF trial compared the factor Xa-inhibitor rivaroxaban (20 mg once daily) to warfarin. In the overall trial, rivaroxaban was similarly effective for prevention of stroke or systemic embolism with a similar rate of major bleeds [22]. In ROCKET-AF 9033 (63.7%) patients had HF. Irrespective of randomisation, embolic events and the rate of major or clinically-relevant non-major bleedings were similar in patients with and without HF. In the 2497 HF patients with reduced LV-EF, rivaroxaban compared to warfarin was associated with numerically lower rates of embolism and numerically higher rates of major or clinically-relevant non-major bleeding (the numbers of major bleed are not publicly reported for this sub-subgroup) [28]. In patients with confirmed HFrEF there is no report on major bleedings alone, which does not allow final conclusions on clinical net-benefit regarding major events.

### Apixaban in ARISTOTLE

The ARISTOTLE trial compared the factor Xa-inhibitor apixaban (5 mg twice daily) to warfarin. In the overall trial, apixaban compared to VKA reduced the rate of stroke or systemic embolism by 21% and the rate of major bleeding by 31% [23]. In ARISTOTLE 3207 patients (22%) had a report of symptomatic HF and an EF > 40% (*n* = 2971) or normal LV systolic function (*n* = 181) or mild dysfunction (*n* = 55) defined as HF with preserved ejection fraction (HFpEF); 2736 (19%) patients had LV systolic dysfunction (LVSD) defined as LV-EF ≤ 40%. Irrespective of randomisation, embolic events were similar in patients with LVSD but tended to be numerically higher in HFpEF compared to non-HF, while the rate of major bleeding according to International Society on Thrombosis and haemostasis (ISTH [29]) was higher in patients with LVSD but similar in HFpEF compared to non-HF. In LVSD patients, apixaban compared to warfarin was associated with lower rates of embolism in the presence of numerically lower rates of ISTH-major bleeding [30]. While the numbers of patients with confirmed HFrEF are still low, the available data suggest a potential clinical net-benefit for apixaban compared to warfarin in HFrEF patients with atrial fibrillation.

### Edoxaban in ENGAGE-AF

The ENGAGE-AF trial compared the factor Xa-inhibitor edoxaban (60 mg once daily) to warfarin. This article will only focus on the higher tested dose of edoxaban (full dose of 60 mg with clinical dose reduction criteria to 30 mg), because another lower dosing regimen of 30/15 mg edoxaban had been tested in another 7034 patients in an original 1:1:1 randomisation, but was not approved for stroke prevention in AF. In the overall trial, edoxaban was equally effective in preventing stroke or systemic embolism (non-significant reduction by 13%) and reduced major bleeding by 20% compared to VKA [24]. In ENGAGE-AF 8145 (58%) patients had HF. Irrespective of randomisation, embolic events were numerically higher in patients with than without HF. In the 3103 HF patients with known LV-EF < 50%, edoxaban compared to warfarin was associated with numerically lower rates of embolism as well as numerically lower rates of major bleeding [31]. The available data suggests a non-inferiority for edoxaban compared to warfarin in HFrEF patients with atrial fibrillation.

The evidence from the four AF trials suggest lower bleeding rates in HF patients on NOACs compared to VKA. Regarding the case scenario for an HF patient in SR, however, they provide no data to justify anticoagulation in this condition.

## Evidence for NOAC to reduce stroke risk in HF with sinus rhythm

While those data are very encouraging for treatment of HF patients with accompanying atrial fibrillation, they cannot be extrapolated to patients in sinus rhythm. Wide-spread off-label use of NOACs should be cautioned as there are observations of lower efficacy of NOACs compared to VKA in resolving LV thrombi once they have occurred [7], while the rate of left-atrial appendage thrombi showed similar resolution on factor Xa-inhibitors compared to VKA [32, 33]. Experimental evidence suggested, that the factor Xa-inhibitor rivaroxaban may have additional haemostatic effects in animal models of HF [34]. However, despite no dedicated trials using NOACs in HF patients in sinus rhythm with equal doses as for stroke prevention in atrial fibrillation have been conducted, some data are available regarding the combination of ASA (100 mg/day) with low-dose rivaroxaban (2.5 mg twice daily).

### Rivaroxaban in HF patients in COMPASS

The Cardiovascular Outcomes for People Using Anticoagulation Strategies (COMPASS) trial compared the factor Xa-inhibitor rivaroxaban at a dose of 5 mg twice daily to ASA (100 mg/day) or the combination of rivaroxaban (2.5 mg twice daily) and ASA (100 mg/day) double-blinded in a 1:1:1 design in 27,395 patients with stable atherosclerotic vascular disease over a mean follow-up of 23 months. Overall, 5902 participants had a history of HF, but patients with advanced HF in New York Heart Association functional class III or IV or having LV-EF < 30% were excluded from this trial [35]. Of the HF patients, 12% had LV-EF < 40% (Table 4). For the subsequent analysis, we will only focus on the ASA/rivaroxaban combination compared to ASA monotherapy. In the overall population, there was a significant reduction of the composite endpoint of cardiovascular death, stroke, or myocardial infarction in HF patients on the combination therapy with some increase in major bleeding. In patients with HF, the stroke rate was 1.4%. Rivaroxaban with ASA reduced the relative risk of stroke (HR 0.48; 95% CI 0.28–0.83) in patients with HF [36]. The effect on stroke reduction appeared interesting, however, the annual mortality rate in this HF cohort ranging about 2–3% is much lower than reported in many HF trials and registries [37, 38]. Furthermore, exclusion of advanced HF patients limits the generalisability of the results also indicated by the lower reduction of stroke risk in patients with reduced LV-EF (Table 5).

**Table 4:** Characteristics of low-dose rivaroxaban trials in HF patients with atherosclerotic disease

	COMPASS [35]	COMMANDER-HF [39]
Comparator	Rivaroxaban vs placebo	Rivaroxaban vs placebo
Background ASA therapy	100 mg/day in all patients	100 mg/day in 93%; 35% on dual-antiplatelet treatment
Rivaroxaban dose	2.5 mg twice daily	2.5 mg twice daily
Patients (*n*) with heart failure	3942 (22% of trial population)	5022 (100%, all patients had HF)
Definition of heart failure	History of HF	History of chronic HF for at least a 3-month
	+ chronic coronary artery disease	+ LV-EF ≤ 40%
	or peripheral artery disease	+ coronary artery disease
	Exclusion of advanced HF defined as either:	+ treatment for an episode of worsening HF within the previous 21 days
	NYHA class III/IV	
	LV-EF < 30%	
Patients with reduced LV-EF	476 (12% of HF population)	5022 (100% of HF population)
Definition of reduced LV-EF	30–40%	≤ 40%
Mean age in HF patients (years)	66	66
Definition of primary safety endpoint	Fatal bleeding, symptomatic bleeding into a critical organ, bleeding into a surgical site requiring reoperation, and bleeding that led to hospitalisation (including presentation to an acute care facility without an overnight stay)	Composite of fatal bleeding or bleeding into a critical space with a potential for causing permanent disability

*ASA* acetylsalicylic acid, *CV* cardiovascular, *HF* heart failure, *LV-EF* left-ventricular ejection fraction, *NYHA* New York Heart Association

**Table 5:** Outcome of low-dose rivaroxaban (2*2.5 mg on ASA background therapy) trials in HF patients with atherosclerotic disease

	COMPASS [35]	COMMANDER-HF [39]
Rate for CV death, myocardial infarction, or stroke on rivaroxaban vs placebo, HR (95% CI)	No HF: 3.8 vs 4.7%; HR 0.79 (0.68–0.93) HF: 5.5 vs 7.9%; HR 0.68 (0.53–0.86)	13.44 vs 14.27/100 p-y; HR 0.94 (0.84–1.05)
Rate for primary safety endpoint on rivaroxaban vs placebo, HR (95% CI)	No HF: 3.3 vs 1.9%; HR 1.79 (1.45–2.21) HF: 2.5 vs 1.8%; HR 1.36 (0.88–2.09)	0.44 vs 0.55/100 p-y; HR 0.80 (0.43–1.49)
Rate for stroke on rivaroxaban vs placebo in patients with reduced LV-EF, HR (95% CI)	2 vs 3%; HR 0.74 (0.23–2.35)	1.08 vs 1.62/100 p-y; HR 0.66 (0.47–0.95)
Rate for major bleeding on rivaroxaban vs placebo in patients with reduced LV-EF, HR (95% CI)	4.7 vs 2.1%; HR 2.30 (0.80–6.62)	2.04 vs 1.21/100 p-y; HR 1.68 (1.18–2.39)

*ASA* acetylsalicylic acid, *CV* cardiovascular, *HF* heart failure, *HR* hazard ratio, *LV-EF* left-ventricular ejection fraction, *p-y* patient-years

### Rivaroxaban in COMMANDER-HF

The Study to Assess the Effectiveness and Safety of Rivaroxaban in Reducing the Risk of Death, Myocardial Infarction, or Stroke in Participants with Heart Failure and Coronary Artery Disease Following an Episode of Decompensated Heart Failure (COMMANDER-HF) was a randomised, double-blind, placebo-controlled trial that compared the factor Xa-inhibitor rivaroxaban at a dose of 2.5 mg twice daily in addition to background therapy (ASA alone or in combination with a thienopyridine was taken by 93% of the patients) in a 1:1 design in 5022 patients who had coronary artery disease, chronic HF with LV-EF ≤ 40%, and had been treated for an episode of worsening HF within the previous 3 weeks (Table 4). Patients were followed-up over a median period of 21 months. The trial’s primary efficacy outcome was the composite of death from any cause, myocardial infarction, or stroke. The primary endpoint occurred with similar frequency in both groups and was mainly driven by all-cause mortality (22.1% vs. 21.8%) [39]. However, on an exploratory basis stroke rate was reduced from 3.0% on placebo to 2.0% on rivaroxaban representing an absolute risk-reduction of 0.54% per 100 patient-years resulting in a number needed-to-treat (NNT) of 185 per year. At the same time, the rate of ISTH-major bleeding increased on rivaroxaban with an absolute risk increase of 0.83% resulting in a number needed-to-harm (NNH) of 120 per year (Table 5) [39].

Patients with HFrEF and CAD are at risk for stroke or TIA in the period following an episode of worsening HF even when AF had not been detected. Most strokes were ischaemic and almost half of them are either disabling or fatal. Rivaroxaban as tested in COMMANDER-HF reduced rates of stroke or TIA compared with placebo by 32% (1.29 events vs. 1.90 events per 100 patient-years, adjusted HR 0.68, 95% CI 0.49–0.94). Fatal bleeding or bleeding into a critical space occurred at a similar rate on rivaroxaban and placebo (0.44 events vs. 0.55 events per 100 patient-years) [40].

An interesting aspect in determining stroke risk in HFrEF patients in sinus rhythm might be detecting new-onset AF. In COMMANDER-HF, new-onset AF was confirmed at study visits in 4.8% of patients during the follow-up. Older age (≥ 65 years), LVEF < 35%, history of PCI or CABG, white race, systolic blood pressure < 110 mmHg, and higher BMI (≥ 25 kg/m^2^) were independently associated with risk of new-onset AF. Anticoagulation with rivaroxaban did not reduce new-onset AF. New-onset AF was associated with a higher risk of subsequent all-cause death (HR 1.38, 95%CI 1.11–1.73). The COMMANDER-HF investigators built a risk score from the variables mentioned above, which could identify patients at risk of new-onset AF [41]. The consequence of detecting AF in HFrEF patients would then be oral anticoagulation with NOAC doses as approved for stroke prevention in AF.

Another potential risk marker for higher stroke risk could be plasma D-dimer levels. Higher plasma D-dimer concentrations HFrEF patients in COMMANDER-HF were independently associated with higher rates of death, stroke, and venous thromboembolism. The all-cause death adjusted hazard ratio of the highest tertile (> 515 ng/mL) vs. the lowest (≤ 255 ng/mL) was 1.77 (95% CI 1.48–2.11; *p* < 0.001). For stroke, patients within the highest D-dimer tertile had the greatest absolute and relative stroke reduction (HR 0.36, 95% CI 0.18–0.70). The number-needed-to-treat to prevent one stroke in the highest tertile was 36 implicating that most of the benefit may be confined to patients with D-dimer concentrations above 515 ng/mL [42].

**Fig. 4:**
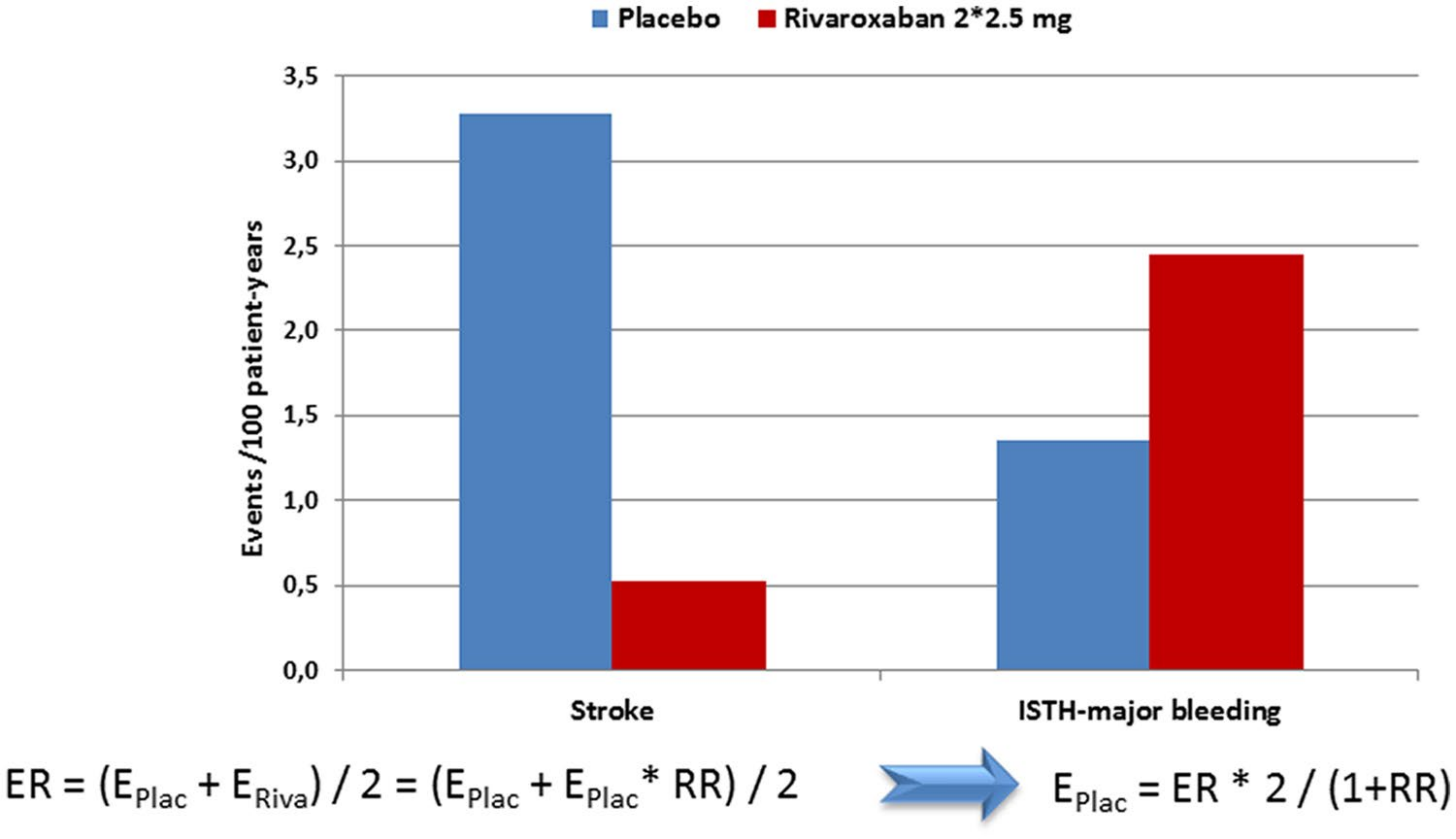
Calculated event rates for stroke and ISTH-major bleedings in patients with cardiovascular disease and heart failure in sinus rhythm in the Western European subgroup of the COMMANDER-HF trial comparing the factor Xa-inhibitor rivaroxaban at a dose of 2.5 mg twice daily to placebo; *ER* event rate of both treatments per region in COMMANDER-HF [43], *E*_*Plac*_ calculated event rate on placebo, *E*_*Riva*_ calculated event rate on rivaroxaban, *ISTH-major bleeding* major bleeding as defined according to the International society on Thrombosis and Haemostasis [29], *RR* relative risk on treatment per region as reported for COMMANDER-HF [43]

A recent report on geographic regions differentially affecting the results in COMMANDER-HF, however, stated the absolute event rates and relative risk by treatment per region. Patients in Western Europe (combined with South Africa) were on average 71-years old, 21% female, had a median NT-proBNP of 2752 pg/mL, and a median LV-EF of 30% with 77% having a LV-EF < 35%. In Western Europe (plus South Africa), the event rates for stroke and for ISTH-major bleeding were reported to be 1.9 per 100 patient-years for both endpoints and the treatment effect of rivaroxaban was reported to be 0.16 for stroke and 1.81 for ISTH-major bleeding, respectively [43]. Based on these data, rivaroxaban reduced stroke rate from 3.3 to 0.5 per 100 patient-years (HR 0.16 (0.04–0.72) while increasing ISTH-major bleeding from 1.4 to 2.4 per 100 patient-years (HR 1.81 (0.61–5.39) (Fig. 4). Therefore, the resulting NNT for Western Europe would be 36/year and the NNH would be 91/year indicating a reasonable net benefit in favour of anticoagulation with rivaroxaban 2.5 mg twice daily.

The evidence from COMMANDER-HF and COMPASS suggest a potential benefit for selected patients with ischaemic HFrEF in SR. Regarding the case scenario for a non-ischaemic HF patient in SR, however, they provide no data to justify anticoagulation in this condition.

## Limitation of current trials

If HF patients have concomitant AF, full-dose NOAC treatment is on-label and subanalyses from the trials as well as meta-analyses indicate that this form of anticoagulation is more efficient in stroke prevention and safer than VKA [26]. In the absence of AF, there are some data for ischemic HFrEF patients for low-dose rivaroxaban plus ASA primarily from the COMMANDER-HF trial [39]. While the risk–benefit-balance both for the composite efficacy endpoint or stroke alone compared to major bleeding was neutral in the overall trial, the net-benefit appears to be in favour of intensified antithrombotic treatment in the Western European subgroup [43]. For patients with non-ischemic HFrEF as in our initial case scenario, we have no trial data available and the overall guideline recommendations are against anticoagulation.

A major limitation when interpreting stroke rates is the differing rate of mortality between trials. When mortality rate is ten-fold higher than stroke rate as in COMMANDER-HF [39] or WARCEF [37], it is difficult to gain a clinically meaningful effect by anticoagulation. If stroke rates are as low as 1% per years, anticoagulation likely induces a relevant rate of major bleedings limiting the overall benefit for the patient despite NOACs showing a more favourable bleeding profile in HF patients than VKA.

## What can we learn from all the trials for a HFrEF patient with CHF in sinus rhythm?

1. All available data indicate a stroke risk in HFrEF patients with sinus rhythm in a range of 1–2%/year. However, given that rather lower stroke risk, which is equivalent to a risk in AF patients with CHA_2_DS_2_-VASc scores of 1–2, none of the trials conducted until now could demonstrate a net-clinical benefit whereby the reduction of stroke risk outweighed the risk of major bleeding.
2. Most NOAC data for HFrEF are available from the large approval trials in AF populations. The only larger NOAC trials in patients with sinus rhythm were conducted with rivaroxaban 2.5 mg twice daily on top of ASA in patients with coexistent vascular disease. Even there, the potential benefit was limited to a sub-group of a sub-group. COMPASS only included LV-EF > 30% and in COMMANDER-HF a potential benefit was merely observed in the Western European population.
3. Whether NOACs given in similar doses as approved for stroke prevention in AF without background therapy with ASA would reduce stroke rates in HFrEF patients with sinus rhythm remains unknown. While this question is clinically relevant, no major trial is addressing it currently.

In summary, there is still no indicator how to optimise anti-thrombotic treatment in the given case of a young HFrEF patient in sinus rhythm with an LV-EF of lower than 30% without co-existing vascular disease. Choice of treatment is still individual and off-label, and guidelines recommend not using any anti-thrombotic treatment.

## Conclusion

In HF patients, stroke risk inversely correlates with LV function even in the absence of atrial fibrillation; however, annual stroke risks are lower in contemporary trials than initially predicted. Routine anticoagulation with VKA did not provide a clinical benefit owing to the increased rate of major bleedings and lower than expected stroke rates. In HF patients with atrial fibrillation, oral factor Xa inhibitors compared to VKA provided at least equally effective stroke prevention, but caused less serious bleeding complications similar to what has been observed in other high risk constellations such as combined antithrombotic therapies [44]. However, major bleedings still occurred in about 2% of patients per year. In the subpopulation with reduced LV-EF, the data for apixaban and edoxaban suggest non-inferiority to VKA in AF patients.

Low-dose rivaroxaban on top of routine ASA treatment in patients with atherosclerotic disease provides promising results. The geographical sub-analysis of COMMANDER-HF suggests that in the presence of contemporary HF therapy, rivaroxaban 2.5 mg twice daily may reduce stroke risk. For HF patients in sinus rhythm without LV thrombus larger trials with stroke/embolic events as primary endpoint are lacking and anti-thrombotic treatment still remains an individual off-label decision even when LV function is severely impaired, which is not recommend by current guidelines.

## Data Availability

Calculated data are available upon request.

## Code availability

Not applicable, no codes used.
